# Intruding implements: a pictorial review of retained surgical foreign objects in neuroradiology

**DOI:** 10.1186/s13244-019-0817-4

**Published:** 2019-12-18

**Authors:** Alessandra D’Amico, Teresa Perillo, Lorenzo Ugga, Renato Cuocolo, Arturo Brunetti

**Affiliations:** Department of Advanced Biomedical Sciences, University of Naples “Federico II”, Naples, Italy

**Keywords:** Foreign bodies, Brain, Spine, Computed tomography, Magnetic resonance imaging

## Abstract

Intra-cranial and spinal foreign body reactions represent potential complications of medical procedures. Their diagnosis may be challenging as they frequently show an insidious clinical presentation and can mimic other life-threatening conditions. Their pathophysiological mechanism is represented by a local inflammatory response due to retained or migrated surgical elements. Cranial interventions may be responsible for the presence of retained foreign objects represented by surgical materials (such as sponges, bone wax, and Teflon). Spinal diagnostic and therapeutic procedures, including myelography, chordotomy, vertebroplasty, and device implantation, are another potential source of foreign bodies. These reactions can also follow material migration or embolization, for example in the case of Lipiodol, Teflon, and cement vertebroplasty. Imaging exams, especially CT and MRI, have a central role in the differential diagnosis of these conditions together with patient history. Neuroradiological findings are dependent on the type of material that has been left in or migrated from the surgical area. Knowledge of these entities is relevant for clinical practice as the correct identification of foreign bodies and related inflammatory reactions, material embolisms, or migrations can be difficult. This pictorial review reports neuroradiological semeiotics and differential diagnosis of foreign body-related imaging abnormalities in the brain and spine.

## Key points

- Intra-cranial and spinal foreign body reactions represent rare medical procedure complications.
- These entities can be incidentally found or investigated due to clinical manifestations.
- Local inflammatory reactions, material embolisms, and migrations are possible pathophysiological mechanisms.
- CT and MRI have a key role in their differential diagnosis.
- Information concerning previous procedure and employed materials is essential as imaging findings depend on material characteristics.

## Introduction

Intra-cranial and spinal foreign body reactions are rare but possible complications of some diagnostic and therapeutic procedures. Due to the frequently insidious onset, nonspecific clinical evolution, and their potential to mimic potentially life-threatening conditions, such as hematomas, neoplasms, radiation necrosis, abscesses, and resolving infarctions, it is important to recognize their imaging findings. These abnormalities can be caused by local inflammatory response either to the post-operative presence of a foreign body in the surgical field or its localization in the central nervous system after mobilization from a different region.

These entities can be incidentally found during exams performed for other indications or in relation to clinical manifestations related to their localization. While first-line imaging exams, such as radiographs, might allow for the detection of some foreign bodies, often other modalities are required for their characterization. Computed tomography (CT) has a high spatial resolution and can detect hyperdense or adipose components, including metallic elements. On the other hand, magnetic resonance imaging (MRI) also allows a more accurate assessment of changes in tissues adjacent to the foreign body. Similarly, contrast agent administration aids in the evaluation of inflammation.

In this review, neuroradiological semeiotics and differential diagnosis of foreign body-related imaging abnormalities in the brain and spine are presented. Infective complications are not included as there is little overlap in imaging signs with reactive alterations.

## Retained foreign objects and related reactions

**Fig. 1.**
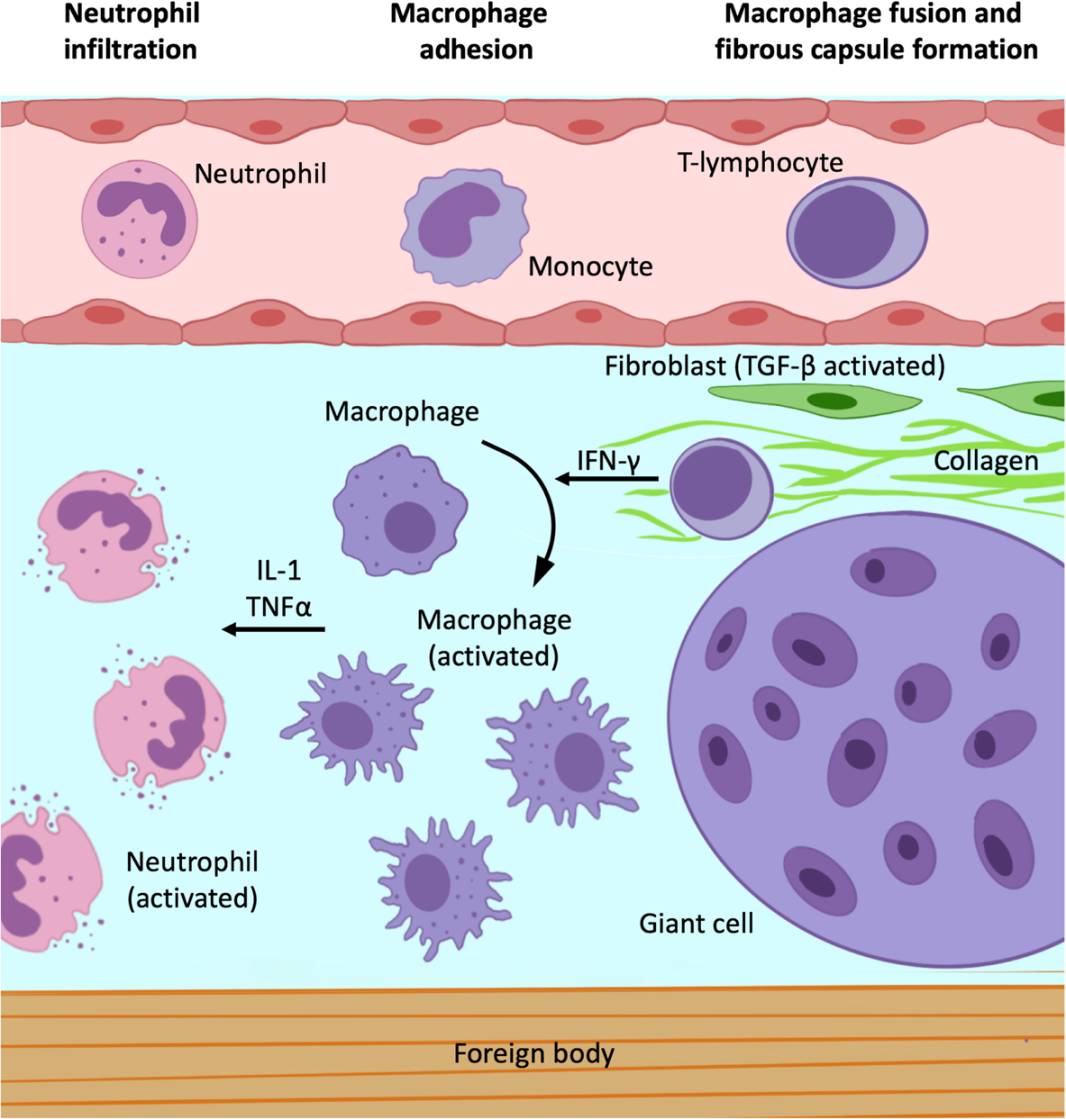
Foreign body granuloma pathophysiology. The foreign body response begins with rapid neutrophil infiltration and subsequent release of soluble factors involved in monocyte circulating monocyte recruitment. These last differentiate into macrophages, which are activated by T cell lymphocytes-produced interferon-γ. In turn, activated macrophages produce interleukin-1 family cytokines and tumor necrosis factor which determine further macrophage activation and their fusion into multinucleated giant cells. After months, transforming growth factor beta releasing recruits and activates fibroblasts with fibrous capsule development

During cerebrospinal surgery, implements made up by different materials may be employed, such as surgical sponges, bone wax, Teflon for suture or micro-vascular decompression, and catheters. Although inert, their intentional or unintentional permanence in the surgical field can lead to inflammatory reactions, especially for non-absorbable materials [1–3]. This aseptic reaction is a form of chronic inflammation, beginning with foreign body retention and consisting of different phases (Fig. 1) [4]. After 6–24 h, neutrophils’ tissue infiltration takes place with subsequent release of soluble regulation factors involved in monocyte extravasation. This occurs after 48–72 h and leads to macrophage activation due to interferon-γ produced by T cell lymphocytes. Macrophages produce interleukin-1 family cytokines and tumor necrosis factor which determine further macrophage activation and their fusion into multinucleated giant cells, which also produce cytokines, in the first weeks, interleukin-1 family cytokines and tumor necrosis factor, whereas after months, transforming growth factor beta is released, with fibroblasts recruitment and fibrous capsule development [5]. Granuloma formation time is extremely variable and can take months or even years [6]. Their appellative also varies based on material composition, for instance, cotton-matrix containing materials determine textilomas or gossypibomas, while gauzomas are caused by gauze.

Granulomas can be localized either intra- or extra-axially. Intra-axial ones appear on CT as well-circumscribed masses with capsular enhancement and a variable amount of vasogenic edema in the surrounding brain tissue. MR shows a mass with heterogeneous signal on T1w, central hyperintensity with peripheral hypointensity on T2w images, and post-contrast ring-enhancement [7, 8]. Extra-axial granulomas appear as a nodular formation, heterogeneously hypointense on T2w images, and with a thin rim of contrast enhancement [9].

Regarding more advanced MRI techniques, granulomas show no increase of relative cerebral blood flow on perfusion maps while a mild increase of the choline/creatine ratio has been reported on spectroscopy. These two features are important in the differential diagnosis with neoplastic lesions [10, 11]. In some cases, they may also show restricted diffusion [12]. When this sign is present, differential diagnosis with abscesses can be challenging, even though these determine symptoms earlier than granulomas [13].

### Surgical sponges

**Fig. 2:**
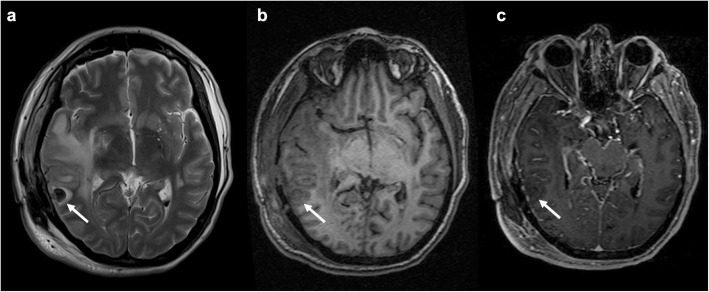
Surgical sponge. Axial T2-weighted (**a**), unenhanced (**b**), and post-contrast (**c**) axial T1-weighted images showing an ovoidal mass (arrow) in the right superior temporal gyrus with low signal on both sequences without enhancement

Surgical sponges are classified as absorbable (gelatin sponge, oxidized cellulose, and microfibrillar collagen) and non-absorbable (cotton or gauze). The latter may present characteristic imaging findings when containing radiopaque filaments. These appear as curvilinear hyperattenuating structures on CT. In MRI, they are characterized by chemical shift artifacts, though not always easily detectable due to the concomitant presence of blood products and air within the surgical area (Fig. 2) [14].

### Bone wax

**Fig. 3.**
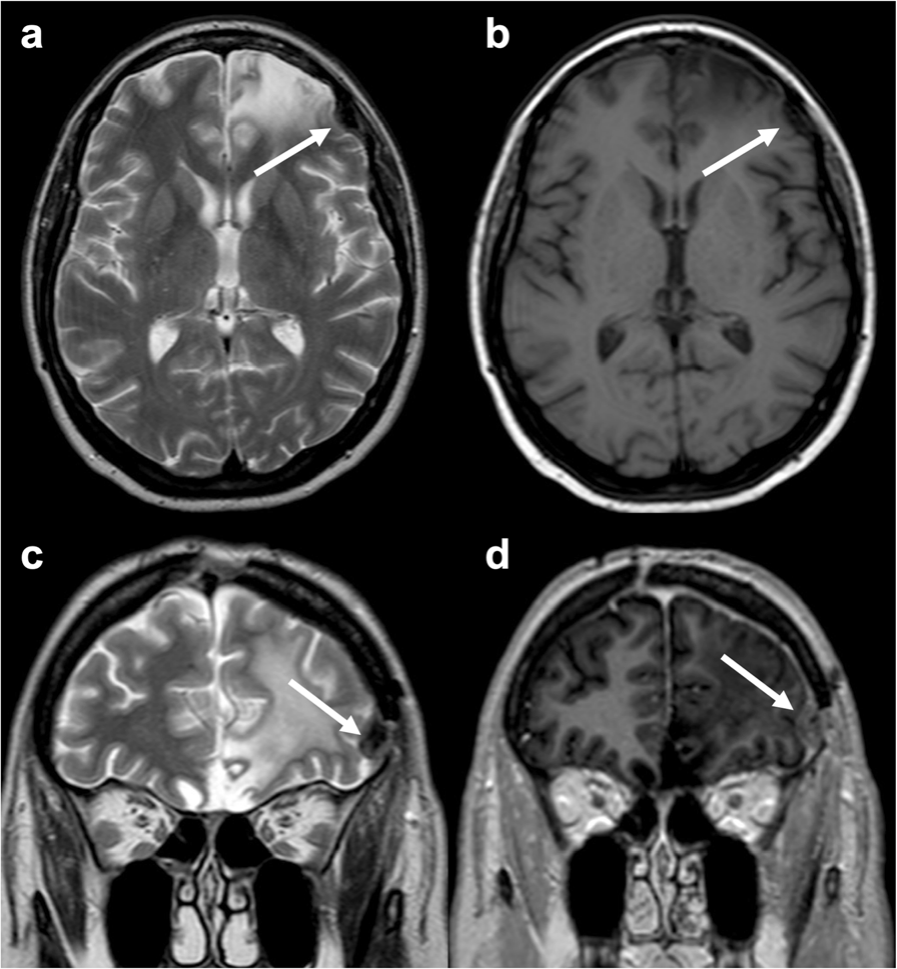
Bone wax. Axial T2-weighted (**a**) and T1-weighted (**b**) and coronal T2-weighted (**c**) and post-contrast T1-weighted (**d**) images depicting burr hole bone wax, hypointense on both T1- and T2-weighted sequences (arrow in **a**, **b**, and **c**). Reactive enhancement is evident on post-contrast T1-weighted image (arrow in **d**)

Bone wax is a mixture of different oils, such as beeswax, Vaseline, and paraffine, and is used to control bleeding from bone surfaces. Even though it is considered safe, in rare cases, it has been associated to chronic inflammation, fibrosis, and granuloma formation [15]. On CT, it presents low density, while on MRI, it produces a signal-intensity void due to its semi-crystalline nature [16]. The two main mimics of bone wax are postoperative air and artifacts from surgical hardware, as they also determine signal-intensity voids on MR (Fig. 3). While postoperative intraventricular and extra-axial air collections are usually easy to distinguish, small intracerebral pneumatoceles may represent a more challenging differential diagnosis. On the other hand, surgical hardware can be recognized by peculiar geometry and artifacts (e.g., bright margins for titanium implants).

**Fig. 4:**
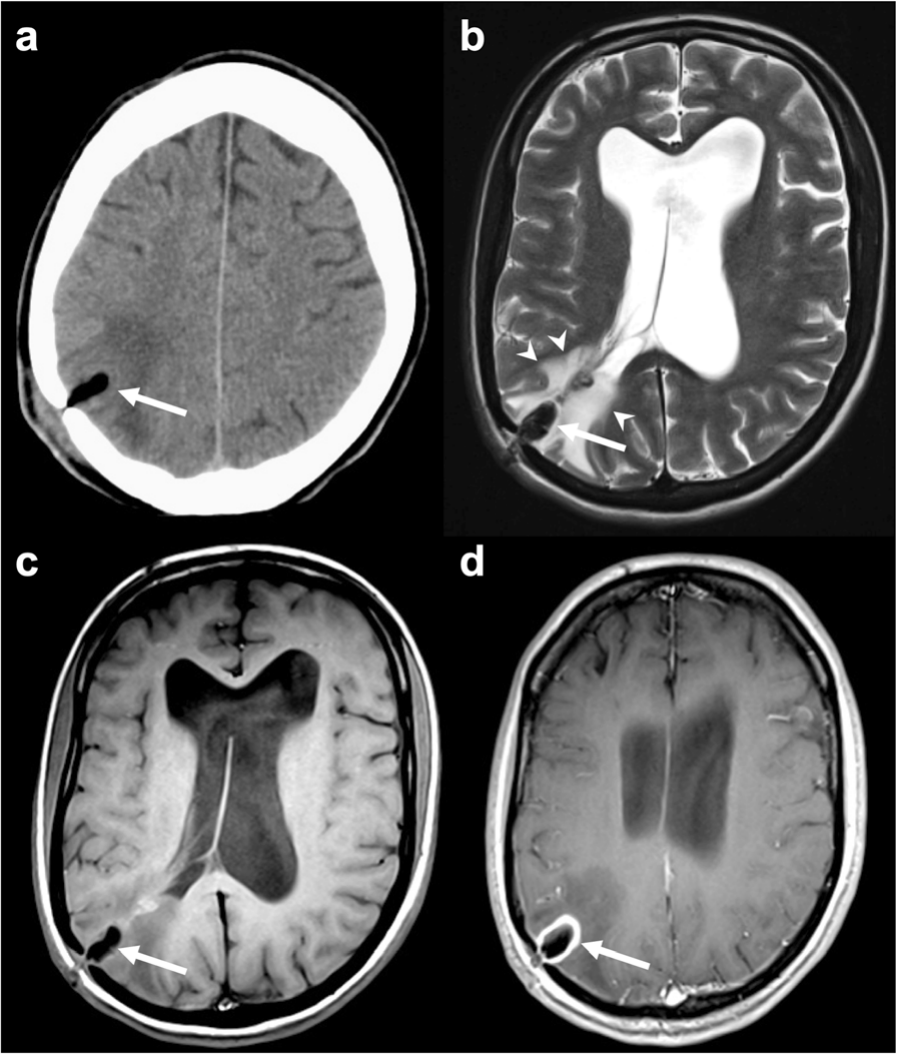
Bone wax granuloma. Axial unenhanced CT (**a**), T2-weighted (**b**), unenhanced (**c**), and post-contrast (**d**) T1-weighted images presenting an ovoidal mass (arrow in **a**, **b**, and **c**) hypointense on all sequences and surrounded by vasogenic edema (arrowheads in **b**). After contrast administration, rim enhancement is detectable (arrow in **d**)

Bone wax granulomas more frequently determine compression on adjacent structures, and, in some cases, the associated hypersensitivity reaction may be particularly aggressive (Fig. 4). For instance, Ateç et al. reported a granulomatous formation extensively infiltrating the medulla oblongata as a consequence of posterior fossa decompressive surgery [1, 17].

### Surgical sutures

**Fig. 5:**
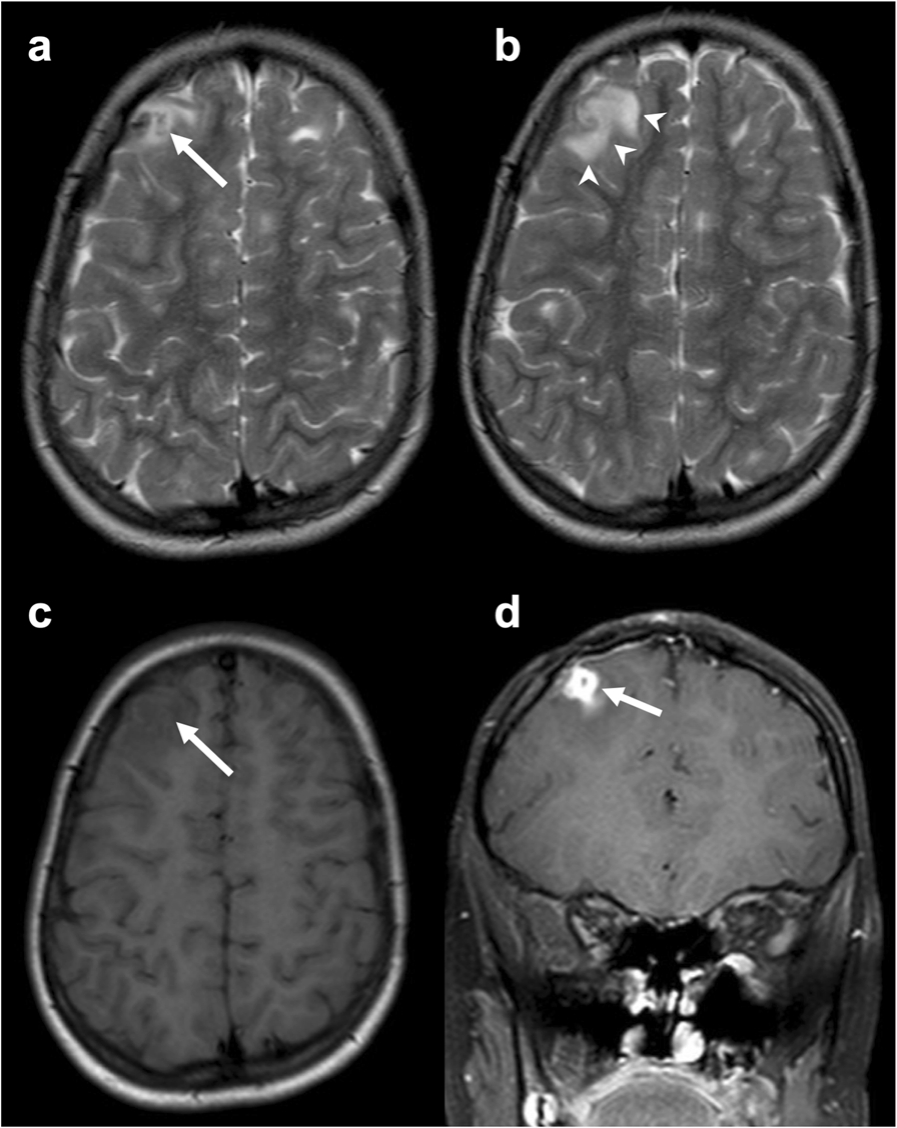
Suture material-induced granuloma. Axial T2-weighted (**a**, **b**) and unenhanced T1-weighted (**c**) images and coronal contrast-enhanced T1-weighted (**d**) showing a mass (arrow) in the right frontal superior gyrus with low signal on both T1- and T2-weighted sequences, surrounded by vasogenic edema (arrowheads), with rim enhancement. Some tubers are detectable in the axial images as the patient was affected by tuberous sclerosis

Surgical sutures can be made using a wide variety of materials, including Teflon, steel wires, silk, and cotton [18]. These are not always detectable, except for steel wires which determine metal-induced artifacts on MRI. Suture-related granulomas appear as well-circumscribed masses with hypointensity on T2w images and enhancement and peripheral vasogenic edema (Fig. 5).

### Teflon

**Fig. 6:**
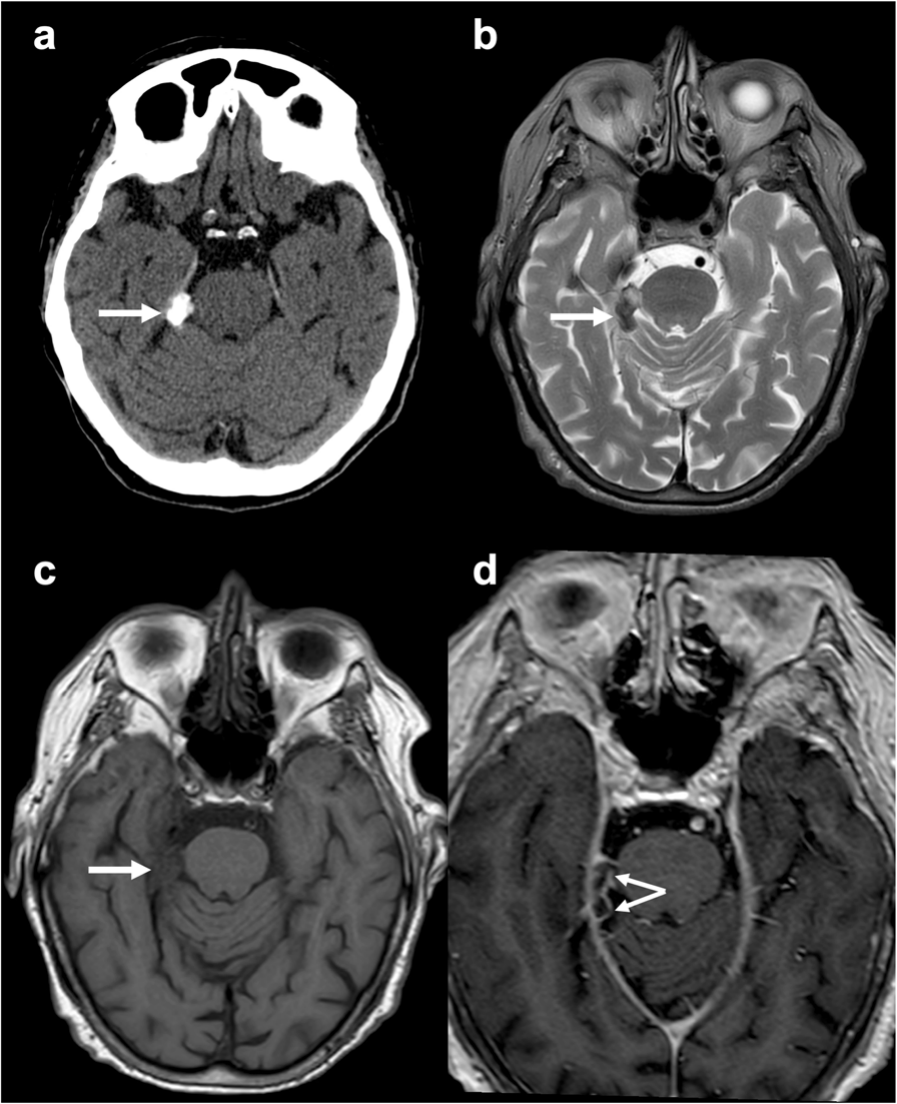
Teflon-induced calcified granuloma. Axial unenhanced CT (**a**), T2-weighted (**b**), unenhanced (**c**) and post-contrast (**d**) T1-weighted images depicting a calcified mass (arrow), with heterogeneous signal and rim enhancement

Teflon is heterogeneously hypointense on T2w images and frequently detectable in the cerebellopontine angle region, as it is commonly used to treat trigeminal neuralgia and hemifacial spasm caused by neurovascular conflict [19]. Unfortunately, a 5% incidence of extra-axial granulomas after its use for micro-vascular decompression has been reported, especially when it comes in direct contact with the dura mater [20]. On susceptibility-weighted imaging, markedly hypointense areas are detectable within the lesion that usually presents extensive calcifications as confirmed by CT (Fig. 6) [9].

### Ventricular shunt

**Fig. 7:**
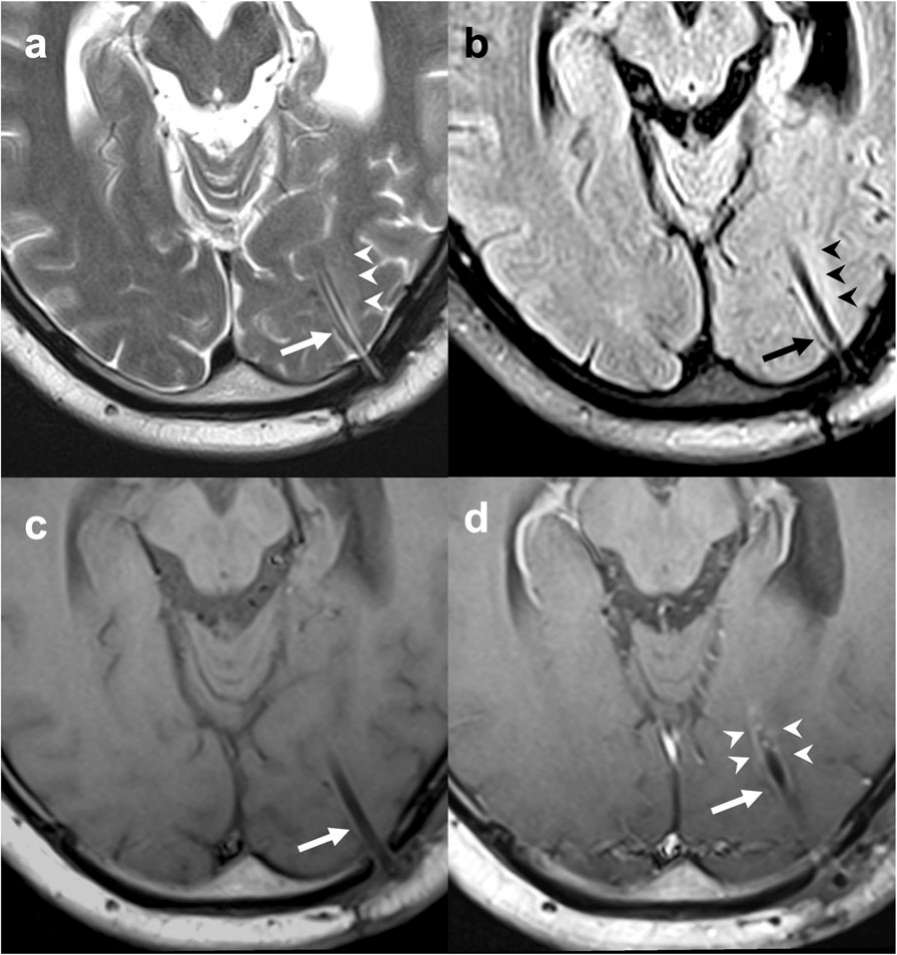
Ventricular catheter. Axial T2-weighted (**a**), FLAIR (**b**), unenhanced (**c**), and post-contrast (**d**) T1-weighted images presenting a left occipital access ventricular catheter (arrows) with mild peripheral gliosis (arrowheads in **a** and **b**) and subtle enhancement (arrowheads in **d**)

Ventricular shunting is the most frequent treatment of hydrocephalus. It is usually performed by external ventricular drain or placement of proximal ventriculostomy and distal peritoneal catheters, a pressure-sensitive valve, and a reservoir. The proximal catheter is easily depicted with both CT and MRI, with peripheral gliosis frequently detectable and possible subtle enhancement (Fig. 7). One of its major complications is mechanical dysfunction which might require surgical revision. Unfortunately, especially for long-time derived patients, it is not always possible to remove the catheter due to high risk of brain damage due to adherences. Therefore, the catheter might be left in situ, increasing the possibility of complications such as abscess and granuloma formation [21]. The latter may assume atypical imaging features, for example resembling a pseudo-tumoral cystic lesion [22].

### Spinal interventions

**Fig. 8:**
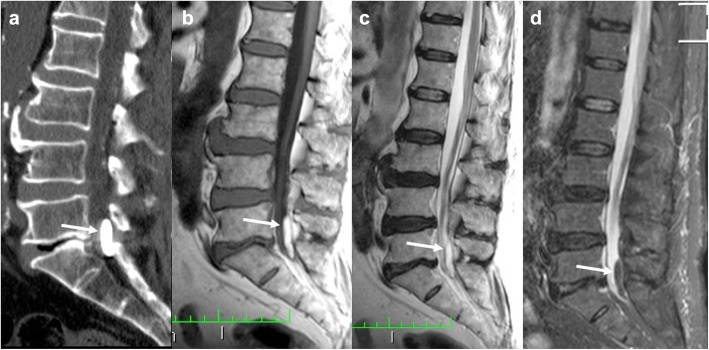
Lipiodol-induced adhesive arachnoiditis. Sagittal unenhanced CT (**a**), T1-weighted (**b**), T2-weighted (**c**), and STIR-weighted (**d**) images showing a mass in the spinal canal at the level of L5-S1 encasing the corresponding nerve roots. It appears hyperdense on CT and hyperintense on both T1- and T2-weighted sequences with signal suppression on STIR (arrows). The cauda nerve roots appear adherent to each other, consistent with the diagnosis of arachnoiditis

Diagnostic and therapeutic spinal interventions may also cause inflammatory reactions and lead to granuloma formation. Adhesive arachnoiditis is a rare complication of myelography and fluoroscopy-guided cordotomy, which were frequently performed with Lipiodol in the past years. It consists of a local inflammation of the arachnoid with adhesive radiculitis. This determines a tethering effect of the spinal cord with subsequent rise of intraspinal pressure and cerebrospinal fluid dynamic alterations, leading to syringomyelia and myelomalacia [23]. Early symptoms resemble acute meningitis. Later, spastic paresis, sensory loss, and incontinence may occur. CT usually shows marked hyperdensity within the spinal canal. On MRI, a mass encasing the spinal cord and nerve roots is usually detected with hyperintense foci due to Lipiodol (Fig. 8) [24]. Syringomyelia and myelomalacia are frequently associated.

Among therapeutic spinal interventions, intrathecal drug therapy is often employed for pain relief in patients who are not responsive to systemic painkillers. Although rare, granuloma formation is possible, in particular when high doses of morphine are used [25].

Vertebroplasty is considered a safe procedure with few complications, even when polymethylmethacrylate leakage occurs. Although rare, it has been associated to radiculitis which could be not only determined by nerve root mechanical compression but also by heat and chemical irritation [26]. Polymethylmethacrylate appears markedly hyperdense on CT, whereas it is hypointense on both T1w and T2w images [27].

Finally, silicon and polyethylene terephthalate-based devices for intravertebral assisted motion, used as a dynamic stabilizer for untreatable back pain, can lead to granuloma formation mostly due to friction caused by the continuous movements of the lumbar spine. On MRI, thecal sac compression, foraminal stenosis, and fluid collections around the device are usually detectable [28].

## Material migrations and embolisms

### Bone wax

Bone wax migration has been described especially after posterior fossa craniotomy where it is used to avoid excessive venous bleeding from emissary veins. When it occurs, a filling defect within the sigmoid sinus both in CT and in MRI is detectable, often extending through the mastoid emissary vein, best depicted with CT and MR angiography. It can often be totally asymptomatic [29, 30]. On the other hand, in some cases, it determines sinus venous thrombosis, especially when massive bleeding of emissary veins occurs during neurosurgery [31]. Spennato et al. have also described a case of bone wax migration into the lateral ventricles of a 2-year-old girl affected by hydrocephalus treated with endoscopic third ventriculostomy. She suffered from recurrent infections which were lately associated to an intraventricular foreign body which has been misdiagnosed as air or a blood vessel [32]. If there are discontinuities within the walls of the frontal sinus, bone wax can also migrate into the orbit with subsequent intraocular pression increase [33]. Moreover, it might also be the cause of late paraplegia in patients who underwent cardio-thoracic surgery, migrating from the surgical area into the epidural space through the inter-vertebral foramina, thus causing spinal cord compression [34].

### Liquid silicon oil

**Fig. 9:**
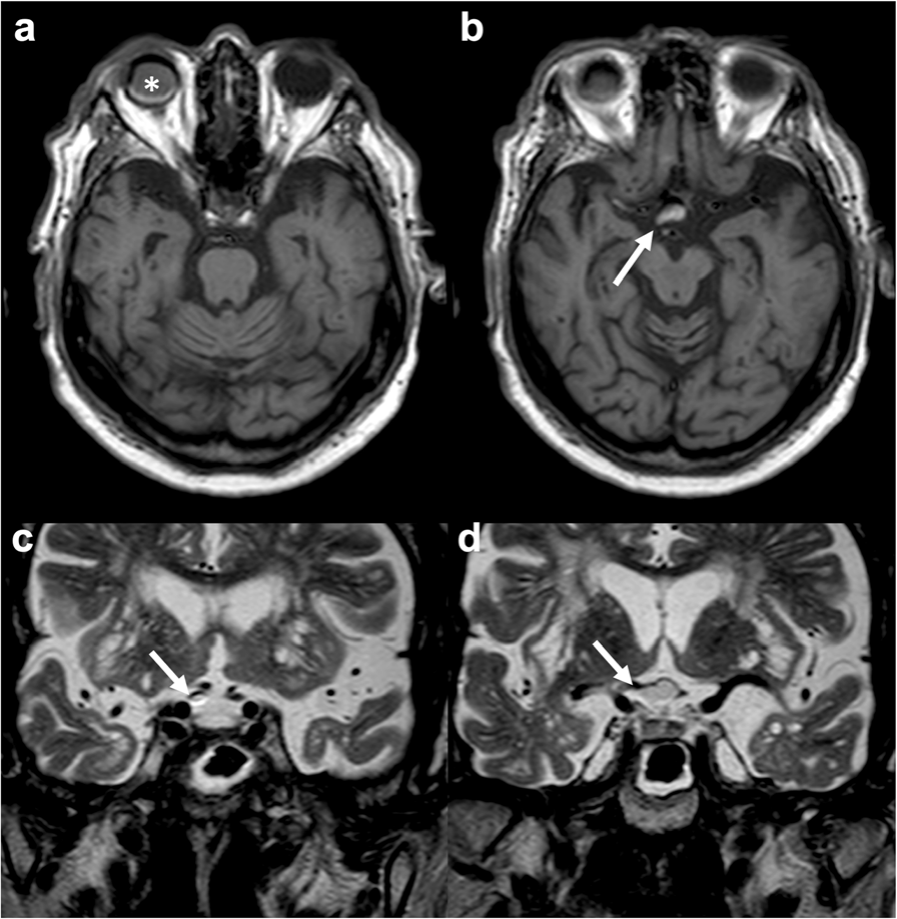
Silicon oil migration. Axial T1-weighted (**a**, **b**) and coronal T2-weighted (**c**, **d**) images demonstrating migration of silicon oil used to treat right retinal detachment (asterisk in **a**) through the right optic nerve sheath up to the right half of the optic chiasm (arrow in **b**, **c**, and **d**). The migrated material has heterogeneous signal also due to chemical shift artifact

Liquid silicon oil is routinely used as an intraocular tamponade agent for retinal detachment treatment. It appears as hyperdense on CT and T1w-hyperintense and T2w-hypointense with a chemical shift artifact on MRI (Fig. 9). On MR spectroscopy, it produces a single peak at 0.33 parts per million [35]. It can migrate from the ocular bulb to the brain through the peri-optic cerebrospinal fluid space, along the optic nerve and chiasm. A rare complication is represented by its migration into cerebral ventricles [36]. In this setting, CT shows intraventricular hyperdense foci which may mimic bleeding. On MRI, silicon droplets are usually easily identified, and a key feature to make the correct diagnosis is their change of position over time [37].

### Lipiodol

Lipiodol is an iodized poppy seed oil which has multiple uses in medicine. It is mostly employed as radiopaque contrast agent or embolic material during procedures like transcatheter arterial chemoembolization or lymphatic embolization. A rare but potentially fatal complication is cerebral Lipiodol embolism. In this setting, CT shows multiple bilateral markedly hyperdense lesions [38]. A rare but specific sign is the “ultradense middle cerebral artery,” consistent with intra-vascular deposition of Lipiodol due to its iodine component [39].

### Cardiovascular procedures

**Fig. 10:**
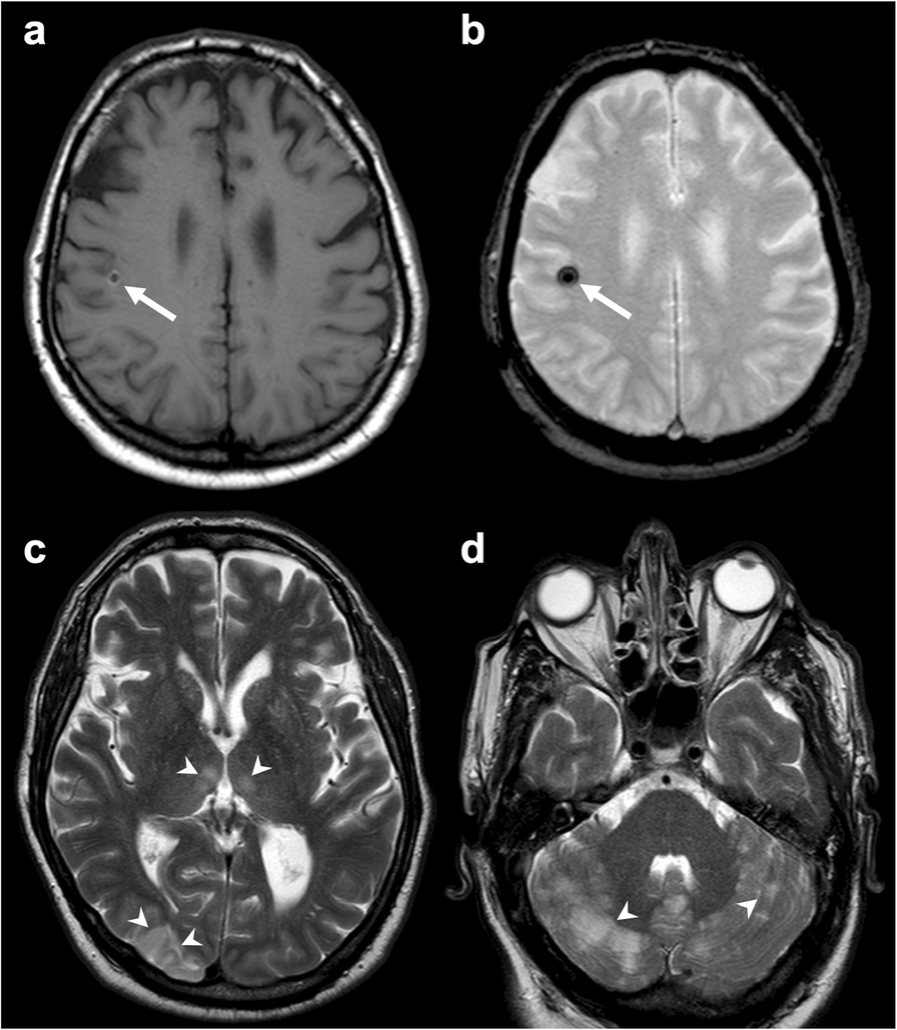
Brain Teflon embolism. Axial T1-weighted (**a**), susceptibility-weighted (**b**), and T2-weighted (**c**, **d**) images depicting multiple ischemic lesions (arrowheads **c**, **d**) due to Teflon emboli (arrow in **a** and **b**) subsequent to aortic dissection surgery

After cardio-thoracic surgery, non-tissue embolism has been reported. From the study of Torre et al., it emerged that 8 of 24 pediatric patients who underwent invasive cardiovascular procedures had foreign body brain embolism. It was associated to different degrees of chronic inflammation. In this setting, cardiac catheters with hydrophilic polymer coating have been identified as the main source of embolism [40]. Emboli can also derive from prosthetic heart valves. On MRI, they appear as multiple punctate susceptibility artifacts in both cerebral hemispheres, due to their metallic nature [41, 42]. Figure 10 depicts an extremely rare case of Teflon embolism subsequent to aortic dissection surgery.

### Vertebroplasty

**Fig. 11:**
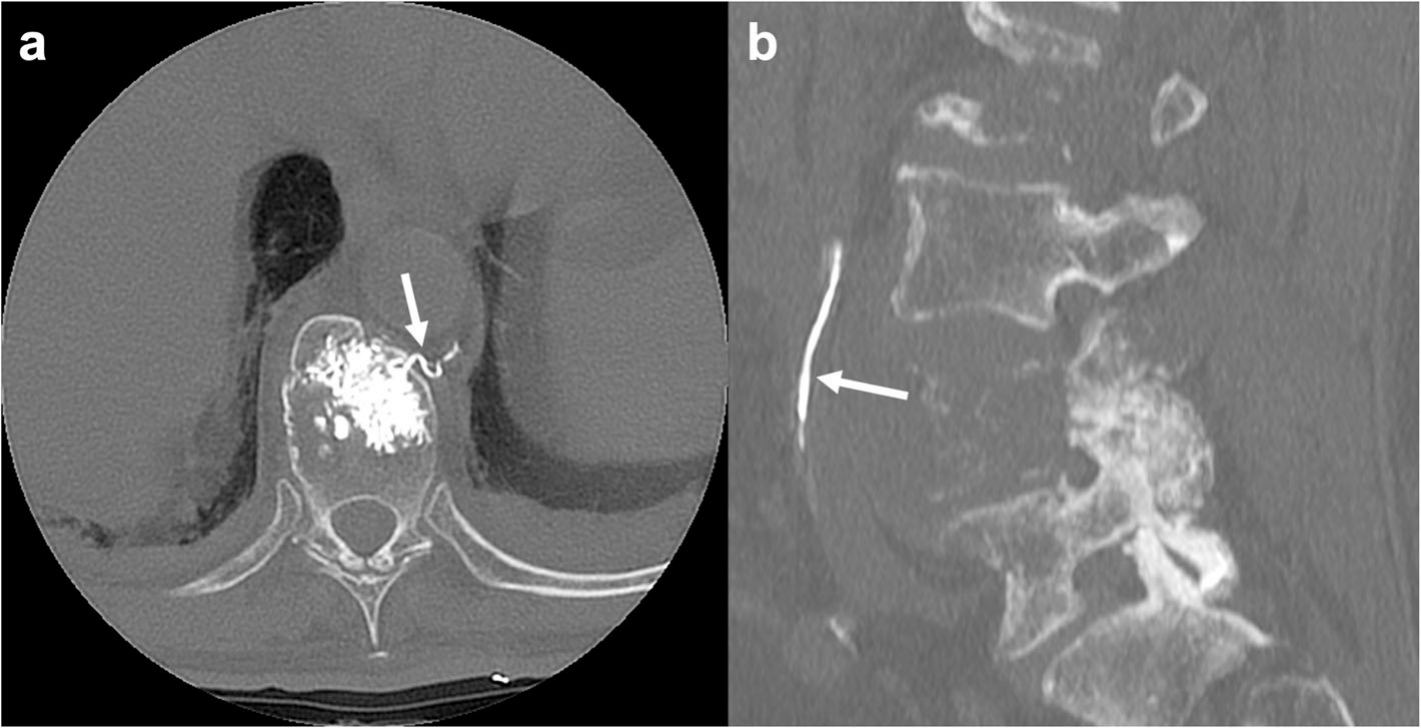
Polymethylmethacrylate migration through veins. Axial (**a**) and sagittal (**b**) unenhanced CT presenting cement migration through an external anterior plexus vein (arrow in **a**) to the inferior vena cava (arrow in **b**)

A possible but rare source of embolism is represented by vertebroplasty (Fig. 11). In these cases, venous passage of cement may determine embolic stroke in the presence of patent foramen ovale or pulmonary arteriovenous malformations [43].

## Differential diagnosis

A thorough knowledge of the patient’s clinical history and the surgical procedures he has undergone is essential to make the correct diagnosis of the above-mentioned abnormalities. However, the differential diagnosis can often be particularly complex, and the use of advanced techniques and controls over time can be useful. Sometimes, a surgical revision may be necessary to resolve the diagnostic doubt. In our opinion, pathologies that more frequently can create differential diagnosis problems are granulomas, chronic adhesive arachnoiditis, and embolism caused by cardiovascular procedures.

Regarding granulomas, the main differential diagnoses are abscesses, hematomas, and tumors. Their radiological features are quite similar, though abscesses may show internal colliquative tissue and air and present restricted diffusion. Hematomas are usually less circumscribed and quite heterogeneous. Tumors are extremely heterogeneous and may show restricted diffusion, increase of relative cerebral blood volume on perfusion maps, and increased choline/creatine ratio at spectroscopy.

The main differential diagnosis of chronic adhesive arachnoiditis is leptomeningeal carcinomatosis. The latter’s key distinguishing feature is its rapid progression, whereas adhesive arachnoiditis may take years to develop.

Embolisms caused by cardiovascular procedures are very rare. They might be suspected when atypical findings are depicted such as metal-induced artifacts caused by cardiac valve material or Teflon.

## Conclusions

Intra-cranial and spinal foreign body reactions are rare but challenging findings as they can mimic other more threatening medical conditions. It is vital for the neuroradiologist to know patient clinical history and especially whether surgical procedures have been previously performed to make the correct diagnosis. Indeed, imaging findings strictly depend on the specific foreign body that has been left in or migrated from the surgical area.

In conclusion, though it may not be easy to recognize foreign bodies and related inflammatory reactions, material embolisms, or migrations, it is important to keep in mind these entities among possible differential diagnoses.

## Data Availability

Not applicable.

## Abbreviations

CT: Computed tomography
MRI: Magnetic resonance imaging
